# Special Issue: Chemoinformatics

**DOI:** 10.3390/molecules21040535

**Published:** 2016-04-22

**Authors:** Peter Willett

**Affiliations:** Information School, University of Sheffield, 211 Portobello, Sheffield S1 4DP, UK; p.willett@sheffield.ac.uk; Tel.: +44-114-2222633

**Keywords:** chemoinformatics, docking, force-field parameter, ligand-based virtual screening, molecular dynamics, molecular similarity, pharmacophore, structure-activity relationships, virtual screening

## Abstract

Chemoinformatics techniques were originally developed for the construction and searching of large archives of chemical structures but they were soon applied to problems in drug discovery and are now playing an increasingly important role in many additional areas of chemistry. This Special Issue contains seven original research articles and four review articles that provide an introduction to several aspects of this rapidly developing field.

Chemistry is, and has been for many years, one of the most information-rich academic disciplines. The very first journal devoted to chemistry was published as early as 1778, and the literature has grown steadily since then. Much of the information in chemistry relates to the structures—in both two and three dimensions—of individual chemical molecules; for example, the world‘s largest chemical database, the Chemical Registry produced by Chemical Abstracts Service, now contains the structures of over 90 million distinct molecules, and there are additional millions in other public databases and in the corporate files of pharmaceutical, agrochemical, and biotechnology companies. This wealth of information has spurred the development of a specialist discipline, that of chemoinformatics, which Gasteiger defines as “the application of informatics methods to solve chemical problems” [1]. Gasteiger has been one of the founding fathers of chemoinformatics, and his review included in this special issue provides a personal view of the history and development of the discipline over the past 50 years. As he notes, the novel modes of access that chemoinformatics provides to even the largest volumes of chemical information have profoundly affected the ways in which chemical research is conducted. That this is so is evidenced by the original research articles and review articles that comprise this special issue, since they provide an overview of current activity in this rapidly developing, and increasingly important field of chemistry.

The structure of a molecule is a prime factor in determining its physical, chemical, and biological properties, and chemoinformatics draws on techniques from areas such as graph theory, multivariate statistics, and machine learning to provide sophisticated data mining facilities to correlate such properties with structure (however this is represented in computational form). Such correlation approaches lie at the heart of virtual screening, which is probably the most important current application of chemoinformatics. Virtual screening involves scanning a database—either of known molecules or of molecules that could in principle be made—to identify those with the greatest probabilities of exhibiting some property of interest, e.g., affecting an individual’s cholesterol level or the viscosity of a lubricant. Several of the research articles in this issue discuss applications of virtual screening. The first two such studies involve the use of similarity approaches. Al-Dabbagh *et al.* report the development of a new family of similarity measures, called Standard Quantum-Based similarity, and describe their use in ligand-based virtual screening using the well-known MDDR, MUV and DUD datasets. Comparable experiments using conventional, fingerprint-based similarity searching demonstrate the effectiveness of the new approach [2]. Shin *et al.* describe an evaluation of four different methods for computing 3D shape similarities. Their experiments used a benchmark dataset based on 22 important therapeutic targets (such as viral pathogens, steroid receptor targets, and GPCRs) and compared the methods in terms of both computational efficiency and screening effectiveness [3].

Two other articles discuss the use of machine learning methods for virtual screening. These methods, such as support vector machine (SVM) or random forest (RF), have become increasingly popular with the availability of greater volumes of structure and activity data for the construction of training sets. Czarnecki *et al.* describe the development of new algorithms—the Extreme Entropy Machine and Extremely Randomized Trees—for predicting bioactivity, and find that they are both more effective and computationally more efficient than SVM and RF, their non-extreme analogues [4]. RFs are also the focus of the study by Li *et al.* [5]. These authors found that, contrary to what might have been expected, the inclusion of low-quality structural and binding data in an RF-based scoring function for a docking algorithm improved the function’s predictive performance.

The remaining research articles cover a diverse range of topics. Mallochi *et al.* report some of the initial results from a long-term project to construct a database of medicinal compounds that provides for each such compound all-atom parameters compatible with different existing biological force fields, microsecond-long dynamics and physico-chemical descriptors in different physiological conditions [6]. Winters-Hilt and Stoyanov discuss the use of an α-hemolysin nanopore transduction detector for a range of applications in biochemistry, biomedical engineering, and biotechnology [7]. Finally, Salmina *et al.* describe an extensive set of manually curated extended functional groups that they have developed for use as descriptors in QSAR and QSPR studies, illustrating the application of these groups to regression and classification tasks on over 20 datasets for which associated physico-chemical or biological property data are available [8].

The review articles are equally wide ranging in character. After providing an overview of pharmacophore modelling and pharmacophore-based virtual screening, Kaserer *et al.* illustrate the use of these techniques with hydroxysteroid dehydrogenases, which are promising therapeutic targets for the treatment of a range of estrogen- and androgen-dependent diseases [9]. Another important biological target is discussed by Kim and Yang, who review the use of structure-based virtual screening in combination with high-throughput screening for the identification of small-molecule inhibitors of hypoxia-inducible factor prolyl hydroxylases [10]. Wang reviews the work of his group and of others on the PI3K/Akt/mTOR pathway, which is a key factor in cellular responses to extracellular stimuli. As Wang notes, while molecular dynamics simulations have long played an important role in drug discovery, they consider only individual molecules; if accurate predictions of biological function are to be achieved then it will be necessary to develop systems dynamics simulations that can model the complex network of interactions between all of the many molecules in a cell [11].

In closing, it is pleasant to note the widespread interest that chemoinformatics is now attracting, with contributions here from Austria, France, Germany, Italy, Malaysia, the People’s Republic of China, Poland, South Korea, the Sudan, Switzerland and the United States of America. I thank all of the authors for their contributions to this Special Issue and the staff members of MDPI for their editorial support.
